# Prenatal HIV Testing in the US-Mexico Border Region, 2005: The Brownsville-Matamoros Sister City Project for Women’s Health

**Published:** 2008-09-15

**Authors:** Christopher H Johnson, Ginger L Gossman, Castrucci Brian C, Kayan L Lewis, Gita G Mirchandani, Carillo Garza Carlos Alberto, McDonald Jill A, Joanna J Nichols

**Affiliations:** Division of HIV/AIDS Prevention, Centers for Disease Control and Prevention; Family Health Research and Program Development, Office of Title V and Family Health, Texas Department of State Health Services, Austin, Texas; Family Health Research and Program Development, Office of Title V and Family Health, Texas Department of State Health Services, Austin, Texas; Family Health Research and Program Development, Office of Title V and Family Health, Texas Department of State Health Services, Austin, Texas; Family Health Research and Program Development, Office of Title V and Family Health, Texas Department of State Health Services, Austin, Texas; Capasits Programma de VIH/SIDA de Matamoros, Tamaulipas Jurisdicción III, Matamoros, Tamaulipas, Mexico; Division of Reproductive Health, Centers for Disease Control and Prevention, Atlanta, Georgia; Texas Department of State Health Services, Health Services Region 8, San Antonio, Texas. At the time of this study, Ms Nichols was affiliated with the Texas Department of State Health Services, Health Services Region 11, Harlingen, Texas

## Abstract

**Introduction:**

Routine prenatal human immunodeficiency virus (HIV) screening provides a critical opportunity to diagnose HIV infection, begin chronic care, and prevent mother-to-child transmission. However, little is known about the prevalence of prenatal HIV testing in the US-Mexico border region. We explored the correlation between prenatal HIV testing and sociodemographic, health behavior, and health exposure characteristics.

**Methods:**

The study sample consisted of women who delivered live infants in 2005 in hospitals with more than 100 deliveries per year and resided in Matamoros, Tamaulipas, Mexico (n = 489), or Cameron County, Texas (n = 458). We examined univariate and bivariate distributions of HIV testing in Matamoros and Cameron County and quantified the difference in odds of HIV testing by using logistic regression.

**Results:**

The prevalence of prenatal HIV testing varied by place of residence — 57.6% in Matamoros and 94.8% in Cameron County. Women in Cameron County were significantly more likely than those in Matamoros to be tested. Marital status, education, knowledge of methods to prevent HIV transmission (adult-to-adult), discussion of HIV screening with a health care professional during prenatal care, and previous HIV testing were significantly associated with prenatal HIV testing in Matamoros, although only the latter 2 variables were significant in Cameron County.

**Conclusion:**

Although national policies in both the United States and Mexico recommend prenatal testing for HIV, a greater proportion of women in Cameron County were tested, compared with women in Matamoros. Efforts between Matamoros and Cameron County to improve HIV testing during pregnancy in the border region should consider correlates for testing in each community.

## Introduction

Human immunodeficiency virus (HIV)/AIDS was first thought to be an acute disease (1,2); however, recent developments in treatment have transformed HIV/AIDS into a chronic condition. The projected life expectancy for those infected with HIV, if they remain in optimal HIV care, has increased from less than 7 years in 1993 to more than 20 years today (1), but optimal care cannot begin without a diagnosis. Routinely testing women prenatally for HIV provides a unique and critical opportunity to diagnose HIV infection, begin chronic care, and prevent mother-to-child transmission.

Perinatal transmission can be prevented in several ways: using antiretroviral drugs for treatment and prophylaxis, avoiding breastfeeding, and electing to have cesarean delivery when appropriate (3-5). Through 2005, 91% of AIDS cases reported among children aged 12 years and younger in the United States were attributed to perinatal transmission (3,4). The number of estimated perinatal HIV infections peaked in 1991 at 1,650 cases and then declined sharply to approximately 142 cases in 2005 (3,6,7). The rate of perinatal transmission is less than 2% with intervention (3,8), compared with 25% to 30% without intervention (3,9).

If a woman is not tested for HIV during her pregnancy, an opportunity for intervention is lost (10). Estimates from the 2004 Pregnancy Risk Assessment Monitoring System (PRAMS) suggest that 87% of women in Texas were tested for HIV during their most recent pregnancy. This figure is higher than the national US estimate of 69% reported in the 2002 National Survey of Family Growth (11). Currently, no estimates are available for prenatal HIV testing in the state of Tamaulipas or for Mexico overall. Very little is known about the prevalence of prenatal HIV testing on the Texas-Tamaulipas border; however, 2.4% of AIDS cases in Mexico are pediatric cases, most of which resulted from transmission from mother to child (12). In 2000, the Mexican National Center for AIDS Prevention (CENSIDA) released an estimate of 0.04% HIV prevalence among pregnant women for 1991 through 1995 (10). A 2003 survey of women tested perinatally in Tijuana found a higher HIV prevalence of 0.65% (12).

Officials from both states, Texas and Tamaulipas, consider the border area to be unique and culturally distinct from the rest of the state or country (13). Border residents share the same cultural identity and are exposed to the same economic conditions, including severe poverty and lack of services (14). The United States and Mexico have engaged in binational collaborations to address the unique public health needs of the border population, including the United States–Mexico Border Health Association and the United States-Mexico Border Health Commission (15,16). The prevalence of prenatal HIV testing in Texas and Tamaulipas does not provide information about prenatal HIV testing on the border. Consequently, the objectives of this study were to 1) report the prevalence of prenatal HIV testing among women who lived in Cameron County, Texas, and among women who lived in Matamoros, Tamaulipas, Mexico, and 2) examine the association between prenatal HIV testing and sociodemographic factors, health behaviors, and health exposures for the sample as a whole as well as for women in each border community. These analyses focused primarily on individual-level characteristics. Other correlates for prenatal HIV testing include national policy and local practice, which were not directly measured.

## Methods

We used primary data collected from 2002 through 2006 from the Brownsville-Matamoros Sister City Project for Women's Health (BMSCP). Brownsville is in the Texas Rio Grande Valley, and Matamoros is a municipality in the state of Tamaulipas, Mexico (Figure). Data from Brownsville were actually collected in all of Cameron County, which is on the southern tip of Texas, adjacent to Tamaulipas. The city centers of Brownsville and Matamoros are only approximately 2 kmapart, and one can easily travel between them by crossing any of 3 bridges that connect the cities.

**Figure.**
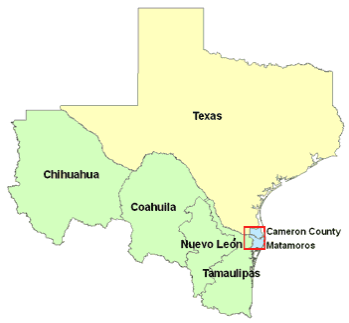
Map of the US-Mexico Border Region and Cameron County, Texas, and Matamoros, Tamaulipas, Mexico (Red Box). (The authors thank Allison Abell Banicki of the Office of Border Health, Texas Department of State Health Services, for creating this map.)

The survey design team selected the sample for the study from among women who delivered live infants on selected days between August 21 and November 9, 2005; participants lived in either Matamoros or Cameron County. Interviewers conducted computer-assisted personal interviews. The Centers for Disease Control and Prevention reviewed this surveillance pilot project for human subjects concerns and determined it to be "nonresearch" or public health practice. Therefore, institutional review board approval was not required.

The sampling design consisted of stratified cluster sampling, and clusters were systematically selected. The survey design team selected all hospitals in each area (Matamoros and Cameron County) that reported 100 or more deliveries per year. Within each hospital stratum, clusters of 2-day blocks were selected. All women who gave birth to a live infant on a selected day, regardless of the time of day, were included in the sample. Interviewers identified eligible participants by periodically reviewing hospital delivery logs and medical records and contacting hospital staff. For a more thorough description of data collection, please see McDonald et al in this issue of *Preventing Chronic Disease* (17).

The survey design team created weights to account for probability of selection, population noncoverage, hospital noncoverage, and nonresponse. We used SUDAAN version 9.03 (RTI International, Research Triangle Park, North Carolina) to account for the complex survey design, which was necessary to allow for appropriate specification of the sampling design parameters.

We used univariate and bivariate distributions for women from Cameron County, women from Matamoros, and the total sample to measure prevalence of HIV testing during pregnancy. The outcome of interest was measured with the question, "At any time during your most recent pregnancy or during delivery, did you have a test for HIV, the virus that causes AIDS?" Correlates of HIV testing were identified by using logistic regression for women from Cameron County, women from Matamoros, and the total sample. In the regressions, the outcome variable was prenatal HIV testing. We selected candidates for the regressions from variables included in the bivariate analyses. Each variable was included in a bivariate logistic regression with the outcome variable. We included only those variables that generated *P* values ≤ .10 in at least 1 place of residence (Matamoros or Cameron County) in the multivariate regressions. Interaction terms were included in a multivariate model for the total sample to confirm the role of place of residence. Variables were not retained in the model if they were not significant at α = .05. Thus, models presented here are parsimonious. The first iteration of regression models included the item, "Were you offered an HIV test during your most recent pregnancy or delivery?" This variable was removed, however, because it was highly correlated with the outcome variable.

Most of the items collected for these analyses were self-reported. The survey design team measured most of the items about HIV, including the outcome measure, by using questions from PRAMS. This team took the item measuring knowledge of HIV infection prevention from Demographic and Health Surveys (DHS) (18) and the Joint United Nations Programme on HIV/AIDS (UNAIDS). The team operationalized knowledge of HIV infection prevention in accordance with the Compendium of Indicators for Evaluating Reproductive Health Programs (http://www.cpc.unc.edu/measure/publications/html/ms-02-06.html). Respondents had knowledge of HIV infection prevention if they reported they could reduce the risk of adult-to-adult transmission by using condoms or having sex only with a single, uninfected partner. The team modified the measure of HIV risk from UNAIDS and DHS. Interviewers read the following item, "I am going to read a list of 3 activities. When I'm done, please tell me if any of the situations apply to you. You do not need to tell me which one. You have used intravenous drugs in the past year; you have been treated for a sexually transmitted disease, sexually transmitted infection, or venereal disease in the past year; you have had multiple (more than 2) sex partners in the past year." The response categories were yes (1 or more applies) or no (none apply).

Although interviewers collected data at hospitals where women delivered, this project used place of residence as the key location variable. Programs and policies in a woman's place of residence are likely to affect her health and pregnancy more than those in the place of delivery, particularly for the outcome of interest, HIV testing during pregnancy or delivery.

## Results

Women in each community were similar in age, employment, and health coverage during pregnancy (Table 1). They varied in terms of education, marital status, and health coverage before pregnancy. A greater proportion of women in Cameron County had a high school diploma or more. A greater proportion of women in Matamoros were either cohabitating or married. A larger percentage of women in Cameron County were without health coverage before pregnancy.

The prevalence of prenatal HIV testing in Matamoros was approximately 37 percentage points lower than the prevalence of HIV testing in Cameron County in 2005 (Table 2). Other notable differences included timing of prenatal care, being offered an HIV test, HIV testing before pregnancy, knowledge of HIV infection prevention, and risk behaviors among women in the sample. A greater proportion of women in Cameron County than in Matamoros entered prenatal care during their first trimester. A larger proportion of women in Cameron County than in Matamoros were offered an HIV test. Although a higher percentage of women in Cameron County were tested for HIV before the reference pregnancy, a higher percentage of women in Matamoros were tested within 6 months before their pregnancy. A greater proportion of women in Cameron County reported 1 of 2 effective methods to prevent HIV transmission, but a larger proportion of women in Matamoros mentioned both condom use and monogamy to prevent transmission.

A greater proportion of women in Cameron County than in Matamoros engaged in behaviors associated with increased risk of HIV before pregnancy. The prevalence of smoking in Cameron County was more than 1.5 times that in Matamoros. The prevalence of drinking and binge drinking in Cameron County was more than twice that in Matamoros. In contrast, the prevalences of intravenous drug use, treatment for a sexually transmitted disease, or multiple partners in the past year were identical for both communities. Smoking and drinking were included in initial regressions but were not significantly associated with HIV testing because they did not vary substantially among women who were tested for HIV.

The bivariate results are category-specific (Table 3 and Table 4). For example, the first row under "age" in Table 3 should be read as "48.6% of the women aged 14 to 19 who lived in Mexico were tested for HIV." The bivariate results show that a greater proportion of women across all categories were tested during pregnancy in Cameron County than in Matamoros. Indeed, there was little variation across categories within Cameron County because of the high testing rate. In terms of demographics, a smaller proportion of cohabitating women in Matamoros were tested for HIV compared with single and married women. Also, a smaller proportion of women in Matamoros with 7 years of education or less were tested compared with women with 8 or more years of education.

In terms of exposure to HIV education, testing, and risk behaviors, the differences between women in Matamoros and those in Cameron County reflected the high prenatal testing rate in Cameron County. Women in Cameron County were consistently tested at higher rates in all categories. Notable differences in Matamoros were seen in the categories for prenatal care, knowledge of effective HIV infection prevention, and exposure to risk. A smaller proportion of women who did not receive prenatal care as early as they wanted were tested for HIV, compared with women who received prenatal care when they wanted. A smaller proportion of women who mentioned condom use were tested compared with women who mentioned monogamy or both condom use and monogamy as an effective prevention method. Finally, only two-thirds of women who engaged in HIV risk behaviors in the previous year were tested during the reference pregnancy.

Of those not tested for HIV during their pregnancy, 91.0% (95% confidence interval [CI], 86.7%-93.4%) lived in Matamoros. Of the women who lived in Matamoros who were not tested for HIV before their pregnancy, 51.3% (95% CI, 46.6%-56.0%) were not tested during the reference pregnancy (compared with 9.6% [95% CI, 6.2%-14.6%] in Cameron County).

The results of logistic regression demonstrated that for the total sample, place of residence, older age, testing before pregnancy, discussion of testing during prenatal care, and knowledge of HIV infection prevention methods were significantly associated with prenatal HIV testing (Table 5). Women who resided in Cameron County were significantly more likely to be tested for HIV prenatally than were women who resided in Matamoros. Women aged 35 to 43 years had nearly 3 times the odds for testing as women in the youngest age group (14 to 19 years). Women who had been tested for HIV before the reference pregnancy had nearly 5 times the odds for testing during the reference pregnancy. Women who discussed HIV testing during prenatal care with a health care professional were significantly more likely to be tested than were women who did not. Finally, mentioning either or both effective HIV infection prevention methods was significantly associated with having been tested for HIV. These results are parsimonious; nonsignificant variables were not kept in the final model.

Three of the variables presented in Table 5 were also significant in the Matamoros-specific regression and behaved similarly (testing before pregnancy, discussion of testing during prenatal care, and knowledge of effective infection prevention) (Table 6). In addition, marital status and education were associated with HIV testing among women who resided in Matamoros. Cohabitation was associated with a lower likelihood of having been tested than was being married. Women with a high school diploma were also more likely to have been tested than were women who had less than a seventh-grade education.

The high prevalence of prenatal HIV testing in Cameron County left little room for variation in the community-specific regression. Only 2 variables were significantly associated with having been tested: having had an HIV test before the reference pregnancy (odds ratio [OR], 5.9; 95% CI, 1.6-21.8; *P* = .01) and discussion of HIV testing during prenatal care (OR, 4.5; 95% CI, 1.8-11.0; *P* = .001).

## Discussion

Prenatal HIV testing was substantially more common in Cameron County than in Matamoros, according to both bivariate and regression analyses. Both also showed that women in Matamoros with a low level of formal education or who were not knowledgeable about adult-to-adult HIV infection prevention were less likely to be tested. Variables associated with prenatal HIV testing did not have the same influence in each community. To confirm these findings, we ran a regression for the total sample that included interaction terms for place of residence with variables that were significant in any of the models, and this regression confirmed the explanatory power of place of residence.

Univariate distributions showed that in some ways these 2 border communities were similar, but in others they varied widely. For example, the distribution of women aged 20 to 34 years was similar in Matamoros and Cameron County, as was the proportion of those employed and the proportion of those who were insured during their pregnancy. However, women varied in their level of education, which may result partly from differences in the educational systems in the United States and Mexico and from differences in socioeconomic status in each community. Women also varied in terms of smoking and drinking alcohol (especially drinking). Alcohol abuse and cigarette smoking are common among people with HIV infection (19), although these behaviors were not significantly associated with prenatal HIV testing in either community.

In addition to individual-level characteristics, public health research must consider policy and practice. In this project, the role of place was probably influenced by national policy regarding prenatal HIV testing. The most recent US policy recommendations state that HIV testing should be offered to all pregnant women as part of standard prenatal tests, regardless of risk factors and prevalence rates in the community (20). The results from this survey suggest the US policy is successful, since nearly 95% of women who resided in Cameron County (and presumably received their prenatal care in their place of residence) were given an HIV test during their most recent pregnancy.

Mexican policy also recommends HIV testing during prenatal care (12); however, the practice of testing varies among insurers. The Mexican health care system is different from the US system in that major insurers also provide services (21), and not all insurers pay for HIV testing. The Mexican Social Security Institute insures employees in the private sector and covers the cost of HIV testing (21). The Social Security Institute for Government Employees, which insures federal employees, also covers the cost of an HIV test. However, the Ministry of Health in Tamaulipas, which serves the uninsured poor, does not cover the cost of an HIV test. An exception is made for high-risk patients, such as injection drug users or people with tattoos (21), but otherwise, women in Tamaulipas covered by the Ministry of Health must pay for their own HIV test.

This policy varies by state in Mexico. Other states, such as Nuevo León, have resources available through the Ministry of Health to pay for widespread HIV testing. In 2007, Mexico initiated a new strategy to use rapid HIV testing for all pregnant women as part of routine prenatal care, with signed consent. Previous policy mandated HIV testing only among women who tested positive for syphilis. However, gauging how this new strategy will affect practice in Mexico will take time.

Our finding of low rates of HIV testing in Matamoros is consistent with other research. One study reported that HIV testing during pregnancy at Tijuana General Hospital is not routinely done (22). Another study reported low rates of testing among high-risk groups in Tijuana and Ciudad Juarez (38% and 30%, respectively) (23). This study also reported a significant association between low education and not having been tested for HIV (23). Our findings are also consistent with previous findings that knowledge of methods to prevent perinatal HIV transmission was associated with HIV testing (23,24).

Findings regarding prenatal HIV testing are mixed in the literature. Previous testing may be a proxy for risk. One recent study suggests that previous HIV testing (ie, knowing one's status) may promote risky behaviors. A study of sex workers in West Africa found that prior HIV testing was associated with decreased condom use (25). In contrast, a study of men who have sex with men found no association between risk (sex and drug use) behaviors and testing during the preceding year (26). In the BMSCP data, previous HIV testing was not significantly correlated with condom use (among those women not trying to get pregnant) or with the measure of HIV risk (intravenous drug use, treatment for sexually transmitted infection, or multiple sex partners) in the total sample or in Cameron County. However, previous HIV testing was correlated with condom use in Matamoros (*P* = .05), where a greater proportion of women who were previously tested for HIV did not use condoms at the time of conception (results not shown).

### Limitations

These data were collected in only 1 border area of 2 sister cities. Although these findings may likely reflect the situation in other border areas and sister cities, they are not necessarily representative of other border areas and sister cities. In addition, the question that measured HIV risk asked only about "intravenous" drug use in the past year. A more precise measure of risk would have asked about "injection" drug use, since some drug users inject intramuscularly or subcutaneously. Also, to maintain validity of data collection, questions asked in both communities were identical, which led to some ambiguity for Matamoros respondents because questions regarding health insurance and Hispanic ethnicity were not entirely applicable. Finally, the BMSCP data do not contain information on where respondents received prenatal care, which would be salient to this research because prenatal care is the most likely setting for an HIV test during pregnancy. US-Mexico border residents often seek health care on the "other side" of the border (27,28). The data contain, however, information on where respondents sought general medical care, and women who reported seeking care either in the nonresident country or in both countries were considered to have crossed the border for care. Seven percent (95% CI, 5.0%-9.7%) of Cameron County residents and 3.6% (95% CI, 2.6%-4.9%) of Matamoros residents reported that they crossed the border for medical care. If many women receive prenatal care across the border, then place of residence may not be the most appropriate measure to use when assessing policy implications.

### Conclusions

Variables that explain prenatal HIV testing are different on each side of the Texas-Tamaulipas border. Only 2 variables were significant in both Matamoros and Cameron County, previous HIV testing and discussion of HIV testing during prenatal care, but the magnitude of the odds and statistical significance varied across the border. Still, this finding shows that HIV testing should be discussed during prenatal care to engage pregnant women in preventive care. The demographic and practice differences between Matamoros and Cameron County are a challenge to health officials because they may preclude sharing intervention strategies across the border. However, practitioners may be able to use the knowledge of these differences to increase testing rates in pregnant patients. HIV testing during pregnancy is essential to minimize mother-to-child transmission and to identify previously undiagnosed cases so that patients may begin chronic care.

## Figures and Tables

**Table 1:** Demographic Characteristics of Women Who Gave Birth in the Texas-Tamaulipas Border Region, 2005: the Brownsville-Matamoros Sister City Project for Women's Health

Characteristic	Country of Residence^a^				Total Sample (N = 947)	

	Mexico (n = 489)		United States (n = 458)			

	n	Weighted % (95% CI)	n	Weighted % (95% CI)	n	Weighted % (95% CI)
**Country of delivery^b^**						
United States	27	5.1 (3.9-6.5)	457	99.8 (98.6-100.0)	484	48.4 (45.5-51.4)
Mexico	462	95.0 (93.6-96.1)	1	0.2 (0.0-1.4)	463	51.6 (48.6-54.5
**Age, y**						
14-19	94	19.2 (16.5-22.3)	68	14.8 (11.9-18.3)	162	17.2 (15.2-19.4)
20-24	154	31.5 (27.8-35.6)	141	30.8 (27.5-34.4)	295	31.2 (28.6-34.0)
25-34	207	42.3 (38.8-46.0)	202	44.0 (40.5-47.7)	409	43.1 (40.6-45.6)
35-39	26	5.3 (3.6-7.6)	39	8.6 (6.2-11.7)	65	6.8 (5.3-8.6)
40-43	8	1.6 (0.9-2.8)	8	1.7 (0.9-3.3)	16	1.7 (1.1-2.5)
**Ethnicity^b^^,^ c ^,^ d**						
Hispanic	489	100.0	394	88.9 (85.5-91.6)	883	95.0 (93.4-96.2)
Non-Hispanic	0	0	49	11.1 (8.4-14.5)	49	5.0 (3.8-6.6)
**Country of birth^d^**						
Mexico	483	99.2 (98.1-99.6)	195	43.2 (38.2-48.3)	678	73.7 (70.5-76.6)
United States	2	0.4 (0.1-1.3)	251	55.5 (50.0-60.9)	253	25.5 (22.5-28.9)
Other	2	0.4 (0.1-1.3)	6	1.3 (0.7-2.7)	8	0.8 (0.5-1.5)
**Marital status^b^^,^ d ^,^ e**						
Single	46	9.4 (7.3-12.0)	119	26.3 (22.7-30.2)	165	17.1 (15.0-19.4)
Live-in significant other	181	37.3 (33.8-41.0)	111	24.6 (21.7-27.6)	292	31.5 (29.1-34.0)
Married	259	53.3 (49.2-57.3)	222	49.1 (45.3-53.0)	481	51.4 (48.6-54.2)
**Education, y^b^^,^ d**						
≤7	154	31.7 (28.4-35.3)	53	11.8 (9.2-14.9)	207	22.6 (20.4-25.0)
8-12 (no high school diploma)	248	51.1 (47.2-55.0)	168	37.4 (32.9-42.0)	416	44.9 (41.8-48.0)
>12 (at least high school diploma)	84	17.1 (13.9-20.9)	229	50.9 (46.1-55.7)	313	32.5 (29.3-35.9)
**Employment status^b^^,^ d**						
Employed	238	49.0 (45.3-52.6)	216	47.9 (42.8-53.1)	454	48.5 (45.4-51.6)
Unemployed	24	4.8 (3.4-6.8)	48	10.7 (7.6-15.0)	72	7.5 (5.8-9.8)
Not in labor force^f^	226	46.2 (42.6-49.7)	186	41.3 (35.8-47.1)	412	44.0 (40.8-74.3)
**Health care coverage^b^^,^ d**						
Before pregnancy						
Coverage	283	58.1 (54.4-61.8)	116	25.3 (21.4-29.5)	399	43.1 (40.2-46.0)
No coverage	206	41.9 (38.2-45.6)	341	74.8 (70.5-78.6)	547	56.9 (54.0-59.8)
During pregnancy						
Coverage	337	69.4 (66.6-72.1)	316	69.2 (65.9-72.4)	653	69.4 (67.2-71.4)
No coverage	151	30.6 (27.9-33.4)	140	30.8 (27.6-34.2)	291	30.7 (28.6-32.8)

Abbreviation: CI, confidence interval.

a Women who reported living in both the United States and Mexico or did not respond to this item were coded as living in the place where they delivered.

b Included in multivariate regression models. Yielded a *P* value ≤.10 in bivariate regression with HIV screening during pregnancy in Matamoros, Cameron County, or total sample.

c All women who reported living in Mexico were coded as Hispanic.

d Frequencies do not add to n's because of missing data. "Don't know" or "refused" responses were included as missing data.

e Women who reported being single, widowed, divorced, or separated were coded as single.

f Homemaker, student, retired, or unable to work.

**Table 2:** Distribution of HIV Testing, Knowledge, and Risk Variables Among Women Who Gave Birth in the Texas-Tamaulipas Border Region, 2005: the Brownsville-Matamoros Sister City Project for Women's Health

Variable	Country of Residence^a^				Total Sample (N = 947)	

	Mexico (n = 489)		United States (n = 458)			

	n	Weighted % (95% CI)	n	Weighted % (95% CI)	n	Weighted % (95% CI)
**HIV testing^b^**						
Had an HIV test during pregnancy						
Yes	260	57.6 (53.5-61.7)	402	94.8 (92.7-96.4)	662	74.7 (71.8-77.5)
No	189	42.4 (38.3-46.5)	22	5.2 (3.6-7.3)	211	25.3 (22.6-28.2)
Timing of most recent HIV test						
1st trimester	87	34.3 (27.8-41.4)	70	17.6 (14.5-21.2)	157	24.5 (21.2-28.2)
2nd trimester	81	31.9 (27.0-37.3)	40	10.2 (7.4-13.8)	121	19.2 (16.4-22.3)
3rd trimester	77	30.1 (25.1-35.7)	59	14.9 (11.2-19.5)	136	21.2 (18.1-24.7)
During pregnancy, but did not know when	5	1.8 (0.9-3.9)	90	22.7 (18.8-27.1)	95	14.0 (11.5-17.1)
During labor/delivery	5	1.8 (0.8-4.4)	136	34.2 (28.0-41.1)	141	20.8 (17.1-25.1)
After delivery	0	0	2	0.5 (0.1-1.8)	2	0.3 (0.1-1.0)
Offered HIV test during pregnancy						
Yes	203	43.7 (40.0-47.4)	394	91.0 (87.8-93.5)	597	65.4 (62.4-68.2)
No	259	56.3 (52.6-60.0)	39	9.0 (6.5-12.2)	298	34.6 (31.8-37.6)
Refused HIV test during pregnancy^c^						
Yes	5	2.5 (1.1-5.6)	4	1.0 (0.5-2.3)	9	1.6 (0.9-2.8)
No	198	97.5 (94.4-98.9)	387	99.0 (97.7-99.5)	585	98.4 (97.2-99.1)
Tested for HIV before pregnancy^d^						
Yes	110	23.5 (19.6-27.9)	237	54.5 (50.3-58.7)	347	37.7 (34.7-40.8)
No	355	76.5 (72.1-80.4)	198	45.5 (41.3-49.8)	553	62.3 (59.3-65.3)
How long before pregnancy was HIV test^d^						
<6 mo	45	41.5 (33.8-49.7)	37	15.8 (11.6-21.3)	82	24.5 (20.3-29.3)
6-12 mo	10	9.0 (4.7-16.6)	50	21.2 (16.4-27.0)	60	17.1 (13.3-21.8)
>1 y	54	49.5 (42.3-56.7)	148	63.0 (56.5-69.0)	202	58.4 (53.4-63.3)
**Prenatal care and HIV information^b^**						
Timing of prenatal care^d^						
1st trimester	217	45.0 (41.4-48.7)	279	62.0 (58.5-65.4)	496	52.8 (50.3-55.3)
2nd trimester	228	47.5 (44.2-50.9)	152	33.8 (30.1-37.7)	380	41.2 (38.7-43.8)
3rd trimester	19	4.0 (2.6-6.0)	16	3.5 (2.5-5.0)	35	3.8 (2.8-5.0)
Did not receive	17	3.5 (2.3-5.2)	3	0.7 (0.3-1.8)	20	2.2 (1.5-3.2)
Prenatal care early as wanted^d^						
Yes	445	91.4 (88.7-93.5)	418	91.5 (89.8-93.0)	863	91.5 (89.9-92.8)
No	36	7.4 (5.5-10.0)	38	8.3 (6.9-10.0)	74	7.8 (6.5-9.3)
Did not want	6	1.2 (0.6-2.6)	1	0.2 (0.0-1.2)	7	0.8 (0.4-1.5)
Discussed getting tested for HIV during prenatal care visits^d^						
Yes	264	55.8 (51.6-60.0)	336	74.4 (67.8-80.1)	600	64.4 (60.6-68.1)
No	208	44.2 (40.0-48.4)	116	25.6 (19.9-32.3)	324	35.6 (32.0-39.4)
Discussed HIV/sexually transmitted disease prevention during prenatal care visits^d^						
Yes	228	48.5 (44.3-52.7)	263	58.2 (52.1-64.0)	491	53.0 (49.5-56.4)
No	243	51.5 (47.4-55.7)	190	41.8 (36.0-47.9)	433	47.0 (43.6-50.5)
**Knowledge of HIV infection prevention^d^^,^ e**						
Mentioned condom use	302	61.7 (56.7-66.5)	297	64.9 (58.5-70.7)	599	63.2 (59.2-66.9)
Mentioned monogamy	15	3.1 (1.7-5.3)	18	3.9 (2.5-6.2)	33	3.5 (2.4-4.9)
Mentioned both	112	23.0 (20.0-26.1)	50	10.9 (8.6-13.8)	162	17.4 (15.4-19.7)
Did not mention either	60	12.3 (8.8-16.8)	93	20.3 (15.8-25.6)	153	15.9 (13.1-19.3)
**Risk factors for HIV infection in past year^d^**						
≥1	28	5.8 (4.4-7.5)	26	5.8 (4.5-7.5)	54	5.8 (4.8-6.9)
None	460	94.2 (92.5-95.6)	426	92.6 (92.6-95.5)	886	94.2 (93.1-95.2)
**Risk behaviors 3 mo before pregnancy**						
Smoked cigarettes^d^^,^ f						
Yes	24	4.9 (3.7-6.5)	36	7.9 (6.2-10.1)	60	6.3 (5.2-7.6)
No	464	95.1 (93.5-96.3)	419	92.1 (89.9-93.9)	883	93.7 (92.4-94.8)
Drank alcohol^d^^,^ g						
Yes	66	13.5 (11.1-16.3)	157	34.5 (31.0-38.3)	223	23.1 (21.1-25.3)
No	422	86.5 (83.7-88.9)	298	65.5 (61.7-69.1)	720	76.9 (74.8-79.0)
Binge drinking^d^^,^ h						
Yes	30	6.2 (4.8-7.9)	59	13.0 (10.2-16.5)	89	9.3 (7.8-11.0)
No	458	93.9 (92.1-95.2)	394	87.0 (83.6-89.8)	852	90.7 (89.1-92.2)

Abbreviations: HIV, human immunodeficiency virus; CI, confidence interval.

a Women who reported living in both the United States and Mexico or did not respond to this item were coded as living in the country where they delivered.

b Frequencies do not add to n's because of missing data. "Don't know" or "refused" responses were included as missing data.

c Reasons for refusing an HIV test included not having money (n = 4), confidence in HIV-negative status (n = 4), and belief the test would hurt the baby (n = 1).

d Included in multivariate regression models. Yielded a *P* value ≤.10 in bivariate regression with HIV screening during pregnancy in Matamoros, Cameron County, or total sample.

e The original item was open-ended. Only those methods described in the Compendium of Indicators for Evaluating Reproductive Health Programs are reported here.

f Respondents who smoked any cigarettes on an average day were classified as smokers.

g Respondents who drank any alcohol were classified as alcohol users.

h Respondents who consumed ≥5 alcoholic drinks in 1 sitting at least once were classified as binge drinkers.

**Table 3:** Percentage of Women Who Had an HIV Test Among Women Who Gave Birth in the Texas-Tamaulipas Border Region, by Demographic Characteristics, 2005: the Brownsville-Matamoros Sister City Project for Women's Health

Characteristic	Country of Residence^a^				Total Sample (N = 662)	

	Mexico (n = 260)		United States (n = 402)			

	n	Weighted % (95% CI)	n	Weighted % (95% CI)	n	Weighted % (95% CI)
**Country of delivery^b^**						
United States	25	100.0	402	95.1 (93.0-96.6)	427	95.4 (93.4-96.7)
Mexico	235	55.4 (51.1-59.6)	0	0	235	55.2 (51.0-59.4)
**Age, y**						
14-19	44	48.6 (41.5-55.8)	55	90.3 (82.6-94.8)	99	64.4 (59.1-69.4)
20-24	75	52.9 (44.6-61.1)	122	93.1 (88.2-96.1)	197	71.3 (65.1-76.7)
25-34	117	62.7 (56.2-68.6)	184	96.3 (93.3-98.0)	301	78.8 (75.0-82.2)
35-39	21	83.8 (63.2-93.9)	34	100.0	55	92.8 (81.2-97.4)
40-43	3	42.2 (14.9-75.4)	7	100.0	10	69.7 (45.2-86.5)
**Ethnicity^b^^,^ c ^,^ d**						
Hispanic	260	57.6 (53.5-61.7)	349	94.9 (92.6-96.5)	609	73.5 (70.4-76.3)
Non-Hispanic	0	0	42	93.1 (77.9-98.1)	42	93.1 (77.9-98.1)
**Country of birth^d^**						
Mexico	258	57.8 (53.7-61.9)	173	95.6 (92.3-97.5)	431	68.0 (64.5-71.3)
United States	2	100.0	221	94.5 (91.6-96.4)	223	94.5 (91.7-96.5)
Other	0	0	5	83.1 (41.1-97.2)	5	60.5 (30.3-84.4)
**Marital status^b^^,^ d ^,^ e**						
Single	26	58.4 (48.2-68.0)	105	95.5 (91.0-97.8)	131	84.2 (78.9-88.4)
Live-in significant other	79	46.5 (40.2-52.8)	94	93.1 (87.4-96.4)	173	62.8 (57.3-68.0)
Married	155	66.0 (60.4-71.2)	200	95.2 (92.4-97.0)	355	79.1 (75.8-82.1)
**Education, y^b^^,^ d**						
≤7	72	49.8 (41.9-57.6)	44	95.7 (85.5-98.8)	116	60.0 (53.2-66.4)
8-12 (no high school diploma)	126	56.6 (49.3-63.5)	141	92.8 (89.0-95.3)	267	70.4 (65.2-75.1)
>12 (at least high school diploma)	61	74.9 (66.9-81.6)	212	96.0 (93.0-97.7)	273	89.9 (86.6-92.5)
**Employment status^b^^,^ d**						
Employed	132	61.2 (55.0-67.0)	193	96.0 (92.5-98.0)	325	77.1 (72.5-81.1)
Unemployed	12	56.3 (36.2-74.5)	44	91.6 (83.3-96.0)	56	80.3 (70.0-87.6)
Not in labor force^f^	116	54.1 (48.6-59.5)	160	94.1 (90.5-96.4)	276	70.9 (67.0-74.5)
**Health care coverage^b^^,^ d**						
Before pregnancy						
Coverage	153	59.0 (52.9-64.8)	104	97.3 (93.3-98.9)	257	69.3 (64.2-73.9)
No coverage	107	55.8 (49.3-62.0)	298	94.0 (91.0-96.0)	405	78.8 (75.2-82.1)
During pregnancy^d^						
Coverage	185	59.8 (55.3-64.1)	279	95.9 (93.3-97.6)	464	76.3 (73.0-79.4)
No coverage	75	52.8 (44.7-60.7)	122	92.4 (87.2-95.6)	197	71.1 (65.8-75.9)

Abbreviations: HIV, human immunodeficiency virus; CI, confidence interval.

a Women who reported living in both the United States and Mexico or did not respond to this item were coded as living in the place where they delivered.

b Included in multivariate regression models. Yielded a *P* value ≤10 in bivariate regression with HIV screening during pregnancy in Matamoros, Cameron County, or total sample.

c All women who reported living in Mexico were coded as Hispanic.

d Frequencies do not add to n's because of missing data. "Don't know" or "refused" responses were included as missing data.

e Women who reported being single, widowed, divorced, or separated were coded as single.

f Homemaker, student, retired, or unable to work.

**Table 4:** Percentage of Women Who Had an HIV Test Among Women Who Gave Birth in The Texas-Tamaulipas Border Region, by Knowledge of and Risk for HIV Infection, 2005: the Brownsville-Matamoros Sister City Project for Women's Health

Variable	Country of Residence^a^				Total Sample (N = 662)	

	Mexico (n = 260)		United States (n = 402)			

	n	Weighted % (95% CI)	n	Weighted % (95% CI)	n	Weighted % (95% CI)
**HIV testing^b^**						
Offered HIV test during pregnancy						
Yes	186	92.0 (87.9-94.8)	377	98.7 (97.2-99.4)	563	96.2 (94.4-97.4)
No	68	28.4 (24.3-32.8)	19	54.2 (40.6-67.3)	87	31.3 (27.1-35.9)
Refused HIV test during pregnancy^c^						
Yes	0	0	3	75.2 (26.5-96.2)	3	31.6 (11.5-62.1)
No	186	94.3 (90.9-96.5)	374	98.9 (97.5-99.6)	560	97.3 (95.8-98.2)
Tested for HIV before pregnancy^d^						
Yes	88	82.8 (75.8-88.1)	225	98.2 (95.1-99.4)	313	93.0 (89.8-95.3)
No	164	48.7 (44.0-53.4)	168	90.4 (85.4-93.8)	332	62.6 (58.7-66.3)
How long before pregnancy was HIV test^d^						
<6 mo	36	85.6 (76.8-91.4)	35	97.1 (84.9-99.5)	71	90.6 (84.5-94.5)
6-12 mo	9	89.7 (61.2-98.0)	48	100.0	57	98.1 (89.8-99.7)
>1 y	42	79.0 (66.8-87.6)	140	97.9 (92.2-99.4)	182	92.4 (87.5-95.5)
**Prenatal care and HIV information^b^^,^ d**						
Timing of prenatal care						
1st trimester	129	65.6 (57.5-72.9)	247	95.0 (91.9-96.9)	376	81.6 (77.2-85.3)
2nd trimester	114	53.8 (48.2-59.3)	136	95.1 (90.6-97.5)	250	69.5 (65.4-73.2)
3rd trimester	12	66.4 (47.1-81.4)	13	100.0	25	79.6 (66.1-88.7)
Did not receive	3	17.9 (7.0-38.7)	1	51.1 (8.0-92.6)	4	21.3 (9.8-40.4)
Prenatal care early as wanted						
Yes	243	59.3 (54.7-63.7)	368	95.1 (92.8-96.7)	611	75.8 (72.7-78.6)
No	14	40.8 (24.3-59.7)	34	94.5 (82.5-98.4)	48	67.0 (54.6-77.4)
Did not want	2	39.1 (8.8-81.1)	0	0	2	33.1 (8.0-73.9)
Discussed getting tested for HIV during prenatal care visits						
Yes	191	77.1 (73.5-80.4)	313	97.5 (95.1-98.7)	504	88.1 (85.9-90.0)
No	66	35.3 (29.4-41.7)	87	87.0 (77.9-92.7)	153	52.1 (45.7-58.4)
Discussed HIV/sexually transmitted disease prevention during prenatal care visits						
Yes	150	70.2 (66.1-74.1)	240	95.3 (92.4-97.1)	390	83.2 (80.4-85.6)
No	106	48.0 (41.9-54.3)	160	94.6 (90.8-96.9)	266	67.1 (62.3-71.7)
**Knowledge of HIV infection prevention^d^^,^ e**						
Mentioned condom use	161	56.3 (51.8-60.4)	271	94.4 (91.8-96.2)	432	74.7 (71.8-77.5)
Mentioned monogamy	10	66.3 (36.5-87.1)	15	94.0 (70.3-99.0)	25	74.4 (70.9-77.6)
Mentioned both	75	70.1 (59.7-78.7)	44	95.7 (85.9-98.8)	119	79.9 (60.6-91.1)
Did not mention either	14	32.7 (21.8-45.9)	72	96.0 (86.1-99.0)	86	77.2 (69.5-83.5)
**Risk factors for HIV infection in past year^d^**						
≥1	18	64.1 (46.7-78.5)	25	100.0	43	80.1 (67.8-88.6)
None	242	57.2 (53.3-61.0)	375	94.5 (92.2-96.1)	617	74.3 (71.6-76.9)
**Risk behaviors 3 mo before pregnancy**						
Smoked cigarettes^d^^,^ f						
Yes	17	73.7 (53.8-87.0)	32	94.1 (81.0-98.3)	49	85.3 (73.9-92.3)
No	243	56.8 (52.5-60.9)	369	94.9 (92.7-96.4)	612	74.0 (71.0-76.7)
Drank alcohol^d^^,^ g						
Yes	40	65.3 (54.8-74.4)	141	93.9 (88.8-96.8)	181	85.1 (79.8-89.2)
No	220	56.4 (51.5-61.3)	260	95.3 (92.3-97.2)	480	71.5 (67.9-74.9)
Binge drinking^d^^,^ h						
Yes	20	68.7 (52.3-81.5)	52	91.2 (82.4-95.8)	72	83.1 (74.8-89.1)
No	240	56.9 (52.2-61.4)	347	95.3 (93.1-96.9)	587	73.8 (70.5-76.8)

Abbreviations: HIV, human immunodeficiency virus; CI, confidence interval.

a Women who reported living in both the United States and Mexico or did not respond to this item were coded as living in the country where they delivered.

b Frequencies do not add to n's because of missing data. "Don't know" or "refused" responses were included as missing data.

c Women stated confidence in HIV-negative status as the reason for refusing an HIV test (n = 3).

d Included in multivariate regression models. Yielded a *P* value ≤.10 in bivariate regression with HIV screening during pregnancy in Matamoros, Cameron County, or total sample.

e The original item was open-ended. Only those methods described in the Compendium of Indicators for Evaluating Reproductive Health Programs are reported here.

f Respondents who smoked any cigarettes on an average day were classified as smokers.

g Respondents who drank any alcohol were classified as alcohol users.

h Respondents who consumed ≥5 alcoholic drinks in 1 sitting at least once were classified as binge drinkers.

**Table 5:** Factors Associated in Logistic Regression With Having Had an HIV Test During Pregnancy, 2005: the Brownsville-Matamoros Sister City Project for Women's Health

**Variable**	OR (95% CI)	*P* value
**Place of residence^a^**		
Matamoros	1.00	
Cameron County	11.65 (6.65-20.42)	<.001
**Age, y**		
14-19	1.00	
20-24	0.75 (0.44-1.27)	.28
25-34	1.35 (0.86-2.12)	.19
35-43	2.74 (1.44-5.22)	.003
**Tested for HIV before pregnancy**		
Yes	4.76 (3.32-7.00)	<.001
No	1.00	
**Discussed HIV testing with health care professional during prenatal care**		
Yes	5.52 (3.89-7.82)	<.001
No	1.00	
**Knowledge of HIV infection prevention methods^b^**		
Mentioned either condom use or monogamy with an uninfected partner	1.82 (1.00-3.31)	.05
Mentioned both methods	2.83 (1.46-5.47)	.002
Did not mention either method	1.00	

Abbreviations: HIV, human immunodeficiency virus; OR, odds ratio; CI, confidence interval.

a Women who reported living in both the United States and Mexico or did not respond to this item were coded as living in the country where they delivered.

b The original item was open-ended. Only those methods described in the Compendium of Indicators for Evaluating Reproductive Health Programs are reported here.

**Table 6:** Factors Associated in Logistic Regression With Having Had an HIV Test During Pregnancy Among Women in Matamoros, 2005: the Brownsville-Matamoros Sister City Project for Women's Health

**Variable**	OR (95% CI)	*P* value
**Marital status^a^**		
Single	0.99 (0.54-1.82)	.98
Live-in significant other	0.57 (0.38-0.84)	.01
Married	1.00	
**Education, y**		
≤7	1.00	
8-12 (no high school diploma)	1.28 (0.78-2.10)	.32
>12 (at least high school diploma)	2.19 (1.19-4.03)	.02
**Tested for HIV before pregnancy**		
Yes	3.56 (2.29-5.55)	<.001
No	1.00	
**Discussed HIV testing with health care professional during prenatal care**		
Yes	6.52 (4.13-9.99)	<.001
No	1.00	
**Knowledge of HIV infection prevention methods^b^**		
Mentioned either condom use or monogamy with an uninfected partner	3.06 (1.48-6.31)	.003
Mentioned both methods	4.30 (2.08-8.90)	<.001
Did not mention either method	1.00	

Abbreviations: HIV, human immunodeficiency virus; OR, odds ratio; CI, confidence interval.

a Women who reported being single, widowed, divorced, or separated were coded as single.

b The original item was open-ended. Only those methods described in the Compendium of Indicators for Evaluating Reproductive Health Programs are reported here.
