# Erratum, Vol 5: Issue 4

**Published:** 2008-12-15

In the article "Prenatal HIV Testing in the US-Mexico Border Region, 2005: The Brownsville-Matamoros Sister City Project for Women's Health," author information for Ginger L. Gossman was omitted. At the time of the study, Dr Gossman was affiliated with the Family Health Research and Program Development, Office of Title V and Family Health, Texas Department of State Health Services, Austin, Texas. The correction was made to our Web site on September 15, 2008, and appears online at http://www.cdc.gov/pcd/issues/2008/oct/08_0106.htm. We regret any confusion or inconvenience this error may have caused.

En el artículo "Realización de la Prueba Prenatal de VIH en la Región Fronteriza México-Estados Unidos, 2005: El Proyecto Para la Salud de la Mujer de las Ciudades Hermanas de Matamoros-Brownsville," se omitió la información de la autora Ginger L. Gossman. Al momento del estudio, la Dra. Gossman estaba afiliada a la oficina de Investigación de Salud Familiar y Desarrollo de Programas, Oficina de Título V y Salud Familiar, Departamento Estatal de Servicios de Salud de Texas, Austin, Texas. La corrección se hizo en nuestro sitio Web el 15 de septiembre de 2008 y aparece en la siguiente dirección de Internet: http://www.cdc.gov/pcd/issues/2008/oct/08_0106.htm. Lamentamos cualquier inconveniente o confusión que este error haya podido causar.

